# A criteria-based progressive rehabilitation program for rectus femoris strain in a recreational soccer player: a case report

**DOI:** 10.3389/fbioe.2024.1385786

**Published:** 2024-08-08

**Authors:** Ángel González-de-la-Flor, Guillermo García-Pérez-de-Sevilla

**Affiliations:** Universidad Europea de Madrid, Faculty of Sports Sciences, Madrid, Spain

**Keywords:** rectus femoris, strain, soccer, rehabilitation, muscle

## Abstract

**Introduction:** The purpose of this study was to describe the criteria-based progressive rehabilitation program implemented on a recreational soccer player diagnosed with a grade 1 rectus femoris strain.

**Methods:** A 33-year-old male injured the rectus femoris muscle. At the first physical examination, the patient showed significant physical impairment due to important limitations to the active range of motion of the knee flexion and the hip flexor strength. The rehabilitation program consisted of specific training of the rectus femoris, lumbopelvic stabilization, mobility exercises, and running technique exercises, for 6 weeks, which was divided into three phases. Each week, the patient performed four rehabilitation sessions, combined with cross-training (swimming), to maintain his cardiorespiratory capacity.

**Results:** The patient improved functionally and returned to play soccer 6 weeks after the injury without pain. Moreover, the patient passed the criteria of each phase at week 2 for phase 1, at week 4 for phase 2 and at week 6 for phase 3.

**Conclusion:** This case study demonstrates that criteria of progression may control the return to sport timetable for recreational soccer players according to the functional and clinical limitations throughout the entire treatment.

## Introduction

Quadriceps muscle injuries are common in sports that require repetitive kicks and sprinting, such as soccer (Ekstrand et al., 2011), and the rectus femoris muscle is the most injured muscle of the quadriceps (Cross et al., 2004). Compared to hamstring or groin muscle injuries, quadriceps muscle strains cause more loss of playing time and higher re-injury rates (Ekstrand et al., 2011).

The rectus femoris plays an important role on the hip and knee joints during kicking (Garrett et al., 1987) and sprinting (Novacheck, 1998) due to its fusiform and biarticular anatomy. This muscle has two proximal heads of origin (direct: antero-inferior iliac spine and indirect: acetabular ridge) and is distally attached to the anterior tibial tuberosity (Hasselman et al., 1995). The muscular actions of the rectus femoris include knee extension, hip flexion, and pelvis stabilization while bearing weight (Shu and Safran, 2011).

Kicking and sprinting are the most common injury mechanisms of the rectus femoris described in the scientific literature (Garrett et al., 1987; Hasselman et al., 1995; Ekstrand et al., 2011). During the acceleration phase of sprinting, especially in the early swing phase, the rectus femoris expands to its maximum length (Riley et al., 2010). At this time, the hip flexor muscles generate force simultaneously as the knee extensor muscles absorb energy through an eccentric action (Schache et al., 2011). In addition, during the ball contact or swing phase of kicking, the rectus femoris may be predisposed to injury due to the stretch-shortening cycle (Brophy et al., 2007).

Several risk factors have been described for the rectus femoris muscle injury, which are divided into intrinsic factors such as age, previous injury, leg dominance, flexibility, or strength (Orchard, 2001) and extrinsic factors such as dry field or temperature (Orchard, 2000). Although several articles, including systematic reviews, have addressed the topic of rehabilitation for rectus femoris injuries, there is still no consensus on the optimal rehabilitation process for this kind of injury. This lack of consensus is present for the management of both recreational and professional athletes. The complexity and multifactorial nature of these injuries necessitate a clear and systematic approach for rehabilitation to minimize re-injury rates (Mendiguchia et al., 2017; Maselli et al., 2021; Ceccarelli et al., 2024).

The purpose of this case report study was to describe the criteria-based progressive rehabilitation program implemented on a recreational soccer player diagnosed with grade 1 rectus femoris strain.

## Case report

### Patient information

A patient was seen at a physical therapy clinic of Comunidad de Madrid. The physical therapy sessions were conducted by an experienced physical therapist with 11 years of clinical practice, starting 5 days post-injury. This case report adhered to the CARE guidelines (Riley et al., 2017). The patient was a 33-year-old male recreative soccer player who described the feeling of being “stabbed” in his thigh while performing sprint training. Earlier that day, he had a feeling of general tiredness. The injury occurred during a running training session, not while playing soccer. Due to a lack of time, the warm-up on the day of the injury consisted only of jogging for 5 min. During the second set of 30-m sprints, while attempting to run at maximum intensity, the patient felt a severe pain in the inner part of his quadriceps and had to stop immediately. The sensation of functional impotence was immediate, and he was barely able to walk. He described his pain as deep, localized, and exacerbated with walking. Using an 11-point numeric pain rating scale, with 0 as no pain and 10 as maximum tolerable pain, the pain was rated 7–8/10.

The patient’s medical history included a previous muscle strain in the same location 3 years earlier. The injury affected his right thigh, which was also his dominant limb, which is essential for strength evaluation. He had been playing soccer recreationally for over 10 years, participating in both weekly matches and training sessions. His regular physical activity included running 10 km, swimming 1,500 m, and strength training for 2 hours each week. He described his pre-injury health status as “very good” and felt he was in an optimal physical condition. This detailed background provides a comprehensive understanding of his fitness level and activity patterns prior to the injury. The patient’s primary goal was to return to recreational soccer.

### Clinical findings and timeline

**FIGURE 1:**
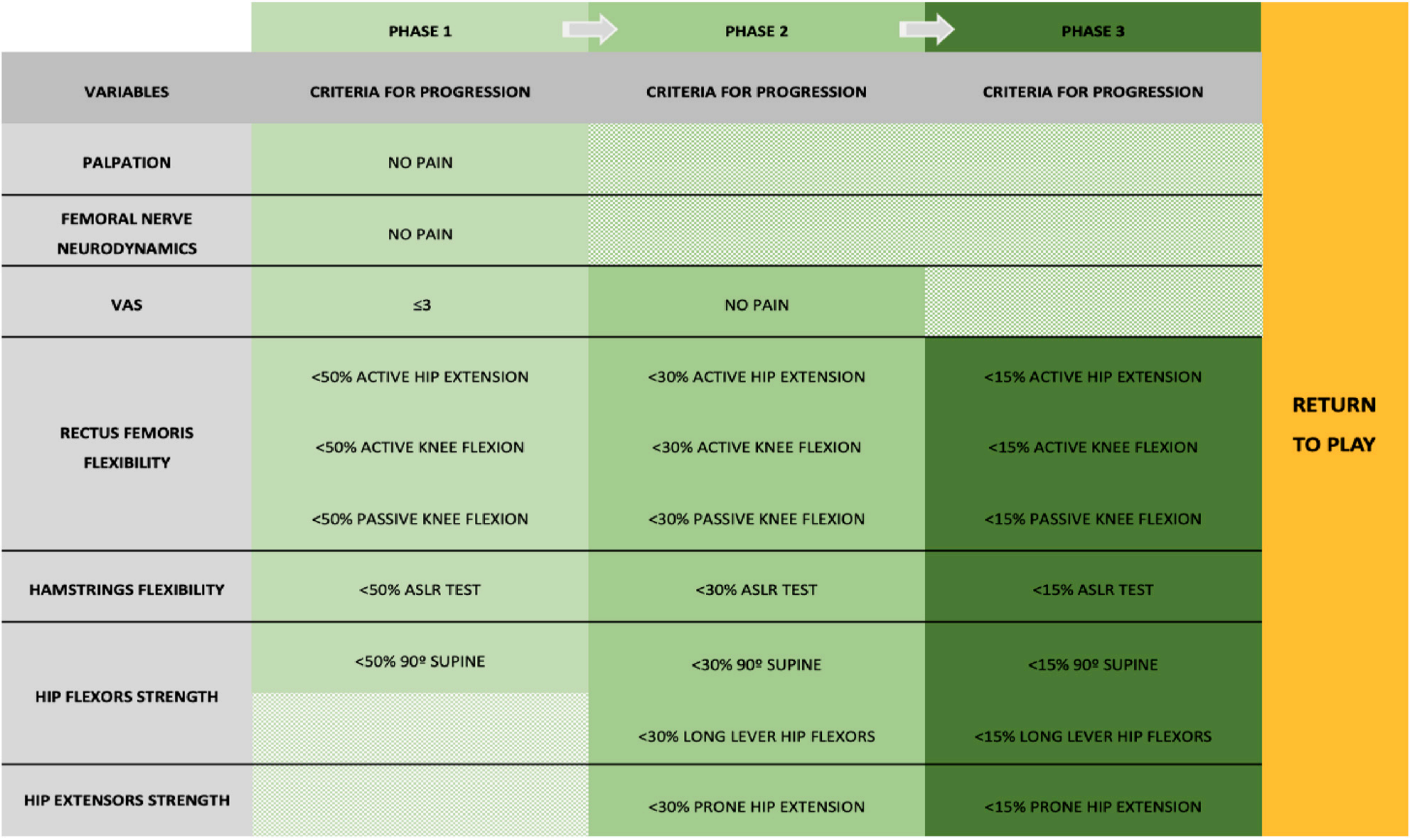
Criteria followed to progress through each phase of treatment.

The initial physical examination and the follow-up (Figure 1) was performed by an 11-year experienced physical therapist. The initial physical examination was performed 5 days post-injury to allow the acute symptoms to stabilize and to obtain a clearer assessment of the injury. Early examination might have been confounded by acute inflammation and pain, potentially leading to an inaccurate diagnosis. This was done in order to rule out other pathologies that cause anterior thigh pain, such as upper-lumbar radiculopathy, femoral neuropathy, and iliopsoas or sartorius muscle injury. The severity of the rectus femoris strain was determined to be grade 1, as confirmed by ultrasonography. This classification aligns with previous research on muscle injuries (Hamilton et al., 2015; Palermi et al., 2021).

**FIGURE 2:**
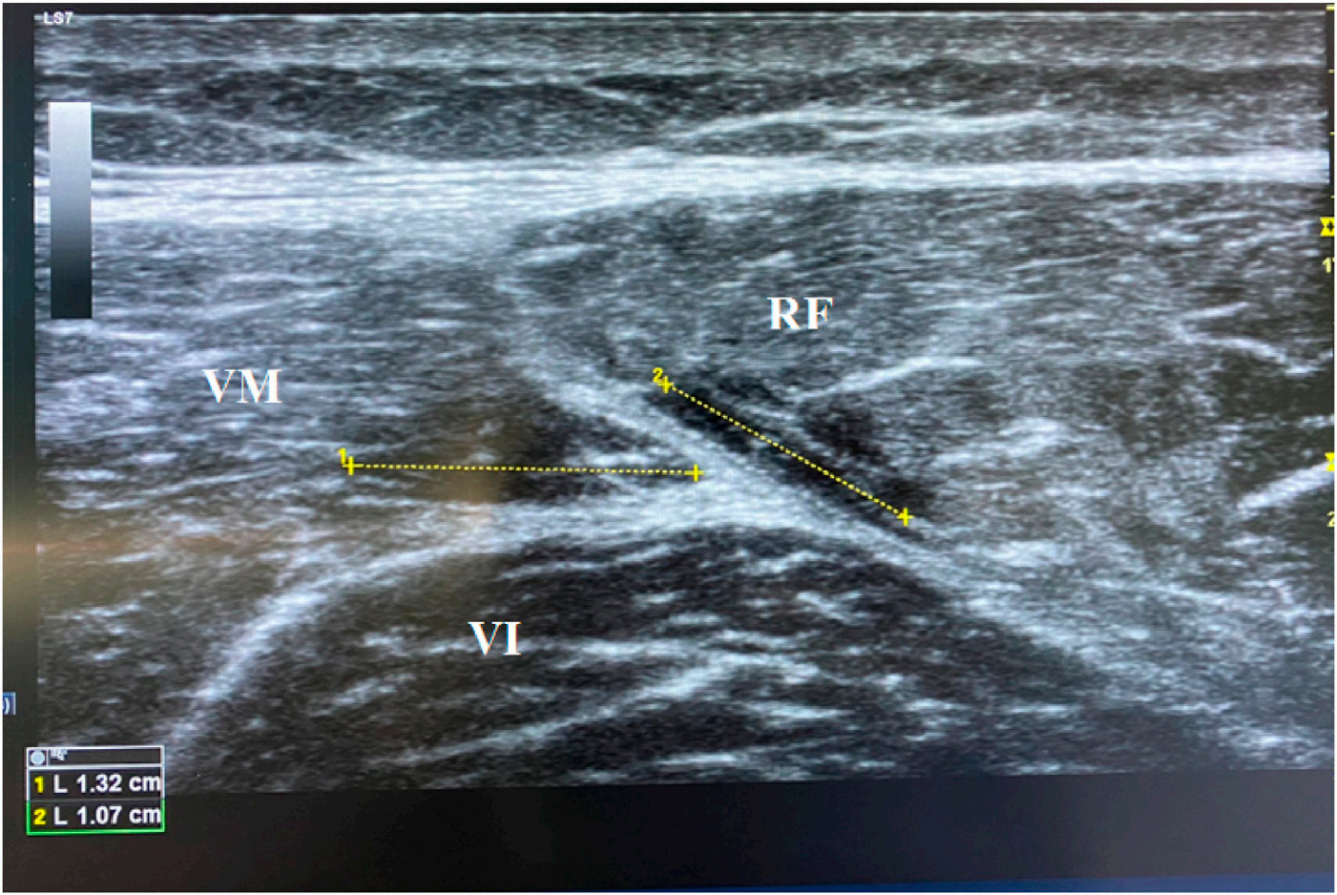
Ultrasonography of the rectus femoris 1 week post-injury.

Figure 2 shows the ultrasonographic image of the rectus femoris 1 week post-injury, indicating the location and extent of the strain. The injury was localized at the mid-belly of the rectus femoris muscle, with no signs of tendon involvement. A final ultrasonographic examination was not performed as the patient had clinically recovered, meeting all functional criteria for returning to play without any reported symptoms.

### Diagnostic assessment

During the physical examinations, pain intensity was consistently evaluated using the visual analog scale (VAS). In phase 1, femoral nerve tension was assessed with the femoral slump test, and active range of motion (AROM) of hip flexion with knee extension was measured through the active straight leg raise test (ASLR) (Liebenson et al., 2009). Additionally, AROM of hip extension with the knee extended in supine decubitus, AROM of knee flexion in prone decubitus, and passive range of motion (PROM) of knee flexion were evaluated (Liebenson et al., 2009). Hip flexor strength at 90 degrees of hip flexion in the supine position and knee extensor strength in the seated position were measured using a digital inclinometer and a hand-held dynamometer (ActivForce 2, USA) (Thorborg et al., 2013). In phase 2, rectus femoris flexibility was assessed by AROM of hip extension, AROM of knee flexion, and PROM of knee flexion, while hamstring flexibility was evaluated using the ASLR test. Hip flexor strength was measured at 90° of hip flexion and at 0° of hip flexion with the knee extended (long lever arm), and hip extensor strength was evaluated in the prone position with knee flexion. In the final phase (phase 3), the same parameters—rectus femoris flexibility, hamstring flexibility, and hip flexor and extensor strength—were reassessed to determine the patient’s readiness to return to play.

The contralateral limb was assessed during the phases 1, 2, and 3 to compare rectus femoris flexibility, hamstring flexibility, and hip flexor and extensor strength between both limbs. The criteria to progress between the phases are described in Figure 1. The physical examination was performed every week to assess all risk factors.

#### VAS

The measurement of the VAS was carried out using a horizontal line that spans from 0 to 10 cm, representing the pain intensity. At one end of the 10-cm line, there was a label indicating the complete absence of pain (0 cm), and at the other end, a label represented the maximum presence of pain (10 cm). The patient indicated their perception or level of pain, which can be directly translated into a numerical score ranging from 0 to 10 (Heller et al., 2016).

#### Femoral slump test

The femoral slump test was used to assess and potentially rule out neuropathy of the femoral nerve. To perform this test, the patient was seated at the edge of an examination table with their legs hanging freely. The examiner instructed the patient to flex their neck forward while keeping their knee extended and ankle dorsiflexed. The examiner then placed one hand on the patient’s shoulder to prevent elevation and abduction of the scapula and the other hand under the patient’s ankle, gently dorsiflexing it. When maintaining this position, the examiner slowly extended the patient’s knee. If the patient experienced pain or paresthesia along the anterior thigh during this maneuver, it may indicate femoral nerve compression or neuropathy (Lai et al., 2012).

#### ASLR examination

The ASLR test was performed to assess the function and strength of the lower trunk and pelvic muscles. During the test, the patient was in a supine position, and they were instructed to lift one of their legs straight while keeping the knee extended. The therapist carefully observed whether the patient could perform this movement without pain or difficulty. Attention was paid to any sensations of tightness, weakness, or pain in the lower back, pelvis, or legs during the leg lift. The ASLR is a common assessment in physical therapy and rehabilitation to evaluate the functionality of the pelvic girdle and lower extremities (Liebenson et al., 2009).

#### ROM examination

The assessment of hip extension and knee flexion PROM and AROM was conducted to evaluate the flexibility of the rectus femoris muscle. During the passive assessment, the patient was in a prone position. The therapist held the patient’s leg by the ankle and performed hip extension while flexing the knee to evaluate the range of motion without the patient’s active involvement. This provided information about the passive flexibility of the rectus femoris. For the knee flexion assessment, both active and passive evaluations were performed. In the passive assessment, the patient was in a prone position, and the therapist gently flexed the patient’s knee joint by bringing the heel toward the buttocks, measuring the ROM without the patient’s muscular effort.

In the active assessment, the patient actively flexed their own knee, trying to bring the heel toward the buttocks, and the therapist observed and measured the ROM achieved through the patient’s voluntary muscle action (Roach and Miles, 1991).

#### Strength examination

The examination of the muscular strength of the hip flexors at 90 degrees of hip flexion, with the hip and knee in extension (using a long lever arm), and the hip extensor (with neutral hip and 90-degree knee bent) were measured (Thorborg et al., 2013).

The patient lied in the supine position, and the hip to be tested was flexed to 90°, while the knee was 90° flexed. The examiner applied resistance just above the knee to assess the strength of the hip flexor muscles at 90° of hip flexion. To assess the hip flexors in a long lever arm, the patient was supine with both the hip and knee fully extended. The examiner applied resistance just above the ankle while the patient attempted to lift the leg off the examination table. Finally, to assess the hip extensor muscles, the patient was in the prone position with a neutral hip position (not flexed or extended) with the knee bent to 90°. The examiner provided resistance just above the ankle, and the patient was asked to raise the thigh off the examination table against the resistance.

### Therapeutic intervention

**FIGURE 3:**
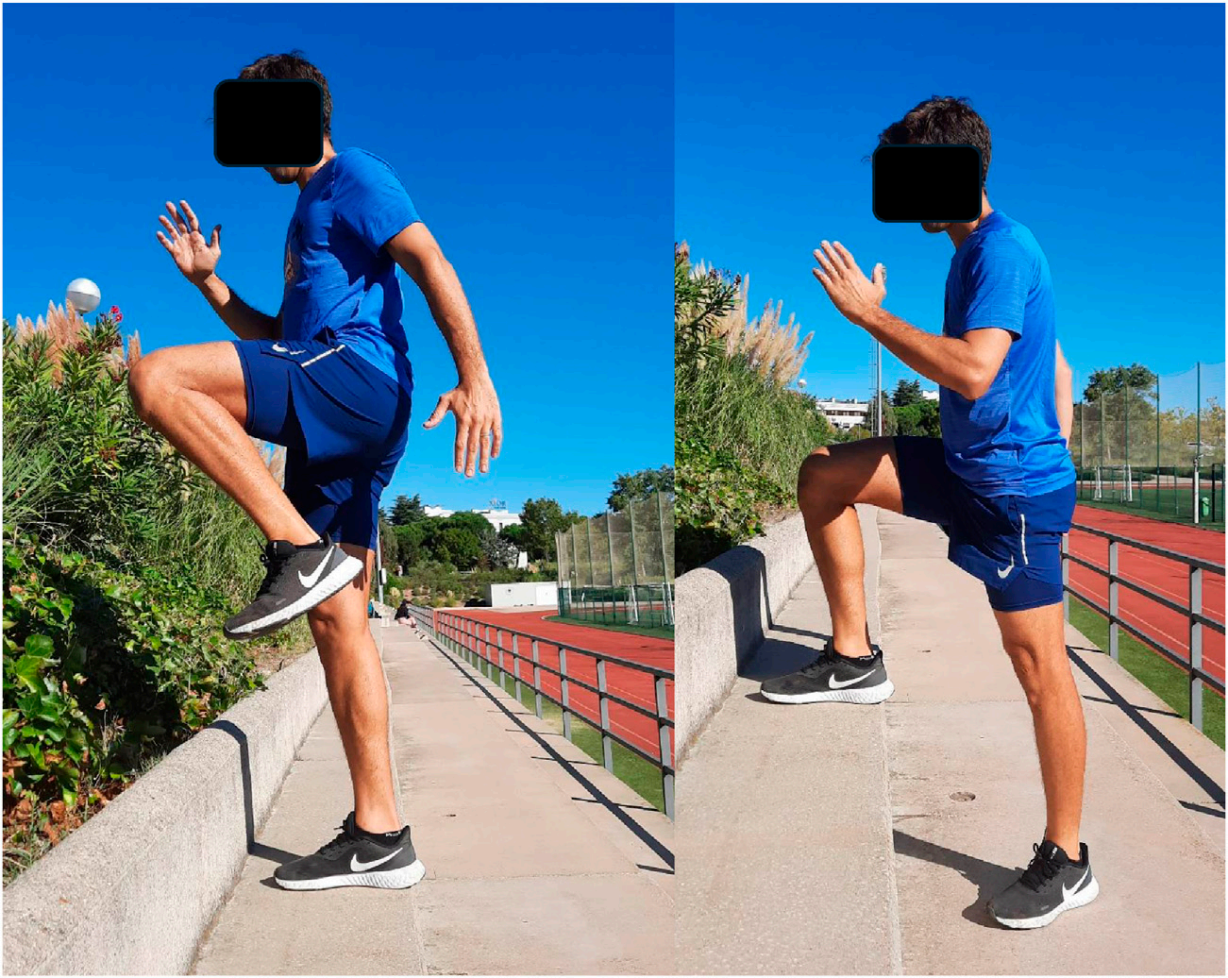
One leg step up exercise.

After the initial clinical examination (5 days post-injury), the recreational soccer player began the rehabilitation program that was controlled by the same physical therapist. The rehabilitation program (Table 1) was adapted from another protocol for hamstring strain injuries, previously published by Mendiguchia et al. (2017) Each week, the patient performed four sessions of rehabilitation and engaged in swimming to maintain cardiorespiratory fitness, which complemented the rehabilitation program by providing low-impact aerobic conditioning. During phase 1 of the rehabilitation program, the patient performed all the exercises (Table 1) adapted according to muscle flexibility, rectus femoris strength, and gluteal strength four times a week (Figure 3). During phases 2 and 3, the rectus femoris flexibility, strength, and gluteal strength exercises were modified, improving the difficulty of the exercises by adding more resistance or with greater multi-joint involvement. The patient was instructed to perform the exercises at an intensity of 8 (very heavy) on the rate of perceived exertion (RPE) scale throughout all three phases of the rehabilitation program.

**TABLE 1:** *Rehabilitation program for rectus femoris injury*.

	Phase 1	Phase 2	Phase 3
**Flexibility**	Prone quadriceps dynamic mobility (2 patterns) 2 × 8 reps Supine hamstring dynamic mobility 2 × 8 reps	Half-kneeling pelvic tilts 2 × 8 reps Hamstring dynamic mobility with fitball 2 × 8 reps	Ballistic swings 2 × 8 reps Half-kneeling pelvic tilts with maximal knee flexion 2 × 5 reps
**Rectus femoris strength**	Isometric supine hip flexion 90° 3 × 5 (3″) reps Standing hip flexion 90° 3 × 5 reps (3″) Leg extension 3 × 6 reps (maximum pain-free weight)	Half-kneeling hip flexion 3 × 5 (3″) reps Inclined trunk (60°) hip flexion 3 × 5 (3″) reps Mountain climbers with slider 3 × 6 reps	Reverse Nordic 3 × 5 reps Walking lunge 4 × 10 m Resisted hip flexion in Thomas test 3 × 4 reps
**Gluteal strength**	**Gluteus medius** Side-lying hip abduction with band 3 × 8 reps Clamshells with band 3 × 8 reps **Gluteus maximus** Bilateral glute bridge (30%BW) 3 × 6 reps Bilateral hip thrust (30%BW) 3 × 6 reps	**Gluteus medius** Lateral walk with band (ankle) 4 × 10 m **Gluteus maximus** Unilateral hip thrust 3 × 8 reps Posterior lunge 3 × 6 reps Step up 3 × 6 reps	**Gluteus medius** Lateral walk with band (feet) 4 × 10 m Monster walk (forward and backward) 4 × 10 m **Gluteus maximus** Plyometric glute bridge 3 × 5 reps Plyometric hip thrust 3 × 5 reps
**Lumbopelvic control**		Side plank 2 × 5 (6″) reps Frontal plank 2 × 5 (6″) reps	Deadbug 3 × 6 reps Pallof press 3 × 5 (3″) reps Frontal plank 2 × 5 (6^a^) reps
**Landing drills**		Step bilateral landing 3 × 5 reps Step unilateral landing 3 × 3 reps Bilateral squat jump 3 × 4 reps	Plyometric jump 3 × 4 reps Bilateral squat (unilateral landing) 3 × 3 reps
**Running drills**		Running 10 m (4 reps) Running 20 m (3 reps)	From skipping to running 10 m (4 reps) High knees running 10 m (3 reps) Butt kicks 10 m (3 reps) Accelerations 10 m (3 reps)

Abbreviations: m, meters; Reps, repetitions; BW, body weight.

The patient followed a 3-day block periodization, where day 1 included running drills and lumbopelvic control, day 2 focused on flexibility and strength training, and day 3 focused on flexibility and lumbopelvic control. During phase 3, the patient performed at least two such treatment blocks per week. Only mild discomfort (VAS ≤ 3) was allowed when performing the exercises. Sets and repetitions of strength training were performed according to previous studies (Mendiguchia et al., 2017).

### Follow-up and outcomes

Outcomes from the patient can be seen in Table 2. Final evaluations were performed by the same therapist who performed the initial evaluations and oversaw each treatment session. Following a program based on specific training of the rectus femoris, lumbopelvic stabilization, mobility exercises, and running technique exercises, the patient improved functionally and returned to play soccer 6 weeks after the injury without pain.

**TABLE 2:** Patient examination findings.

** **	1 week post-injury	2 weeks post-injury	3 weeks post-injury	4 weeks post-injury	5 weeks post-injury	6 weeks post-injury	Return to play	12 weeks post-injury
**VAS**	8	3	0	0	0	0		0
**Femoral nerve neurodynamic test**	**+**	**-**	**-**	**-**	**-**	**-**		**-**
**ASLR test**	Right 97.24° Left 97.91°	Right 97.19° Left 101.62°	Right 113.36° Left 100.32°	Right 111.29° Left 108.73°	Right 114.18° Left 108.08°	Right 112.27° Left 108.55°		Right 106.36° Left 99.26°
**Hip extension knee extended AROM**	Right 33.56° Left 34.75°	Right 33.06° Left 33.75°	Right 35.56° Left 35.09°	Right 35.3° Left 34.21°	Right 33.12° Left 32.46°	Right 34.27° Left 32.98°		Right 35.21° Left 34.53°
**Knee flexion AROM**	Right 101.72° Left 132.09°	Right 106.76° Left 130.78°	Right 119.31° Left 128.88°	Right 117.03° Left 128.89°	Right 123.29° Left 128.8°	Right 127.86° Left 134.78°		Right 141.09° Left 133.47°
**Knee flexion PROM**		Right 110.39° Left 151.04°	Right 127.9° Left 153.08°	Right 138.32° Left 156.69°	Right 147.93° Left 151.19°	Right 150.45° Left 148.05°		Right 148.85° Left 155.58°
**Hip flexor strength** **(90° hip flexion, knee flexed)**	Right 37,35 N Left 195,53 N Difference: 81%	Right 101,99 N Left 191,69 N Difference: 48%	Right 149 N Left 196,23 N Difference: 24%	Right 170,18 N Left 233,68 N Difference: 27%	Right 175,35 N Left 218,97 N Difference: 20%	Right 175,26 N Left 230,39 N Difference: 14%		Right 214,91 N Left 253,61 N Difference: 15%
**Hip flexor strength** **(0° hip flexion, knee extended**			Right 86,69 N Left 131,08 N Difference: 34%	Right 117 N Left 162,1 N Difference: 28%	Right 131,2 N Left 169,47 N Difference: 23%	Right 150,97 N Left 170,85 N Difference: 12%		Right 136,61 N Left 137,55 N Difference: 1%
**Hip extensor strength** **(0° hip flexion, knee flexed)**			Right 131,15 N Left 167,02 N	Right 196,71 N Left 175,97 N	Right 221,76 N Left 230,9 N	Right 291,17 N Left 290,8 N		Right 283,14 N Left 251,98 N
**Knee extensor strength** **(sit down, 90° hip flexion, 90° knee flexion)**	Right 340,62 N Left 354,64 N	Right 372,36 N Left 383,2 N	Right 378,57 N Left 395,02 N	Right 382,4 N Left 400,34 N	Right 394,44 N Left 415,15 N	Right 439,72 N Left 466,46 N		Right 468,2 N Left 464,2 N

Abbreviations: AROM, active range of movement; PROM, passive range of movement; VAS, visual analogic scale; ROM, range of motion; °, degrees; N, newton.

The patient reported pain when performing his daily tasks only during the first 2 weeks after the injury and was able to run at 10 km/h for 10 min 25 days after the injury. The criteria to return to run were having rectus femoris and hamstrings flexibility and a hip flexor and extensor strength deficit of <30% compared to the contralateral (Mendiguchia et al., 2017) (Figure 1).

Improvements were observed in the right knee flexion range of motion and the right hip flexor strength throughout the 6 weeks of treatment and then in the 12 weeks post-injury follow-up. In addition, the hip and knee extensor strength of both limbs improved throughout the 6 weeks of treatment and was maintained in the 12 weeks post-injury follow-up. Moreover, at 12 weeks post-injury, clinical deficits between limbs in hip flexion strength at 90° of hip flexion persisted. At discharge 6 weeks after the injury, the patient achieved his goal of returning to play recreational soccer. Moreover, the patient passed the criteria of each phase at week 2 for phase 1, at week 4 for phase 2, and at week 6 for phase 3 (return to sport).

## Discussion

This case report describes a novel criteria-based rehabilitation program for the rectus femoris strain. Previous studies have proposed rehabilitation protocols for similar muscle injuries, but few have detailed criteria-based progression for the rectus femoris specifically. The recovery and return to play within 6 weeks align with the timelines reported in the literature for similar injuries, suggesting the efficacy of our treatment approach (Bogwasi et al., 2023). During sprinting, the rectus femoris lengthens and generates forces simultaneously as the knee extensor muscles absorb energy through an eccentric action (Schache et al., 2011).

Several studies have proposed rehabilitation protocols for muscle injuries, but few have detailed criteria-based progression specifically for muscle injuries (Mendiguchia et al., 2017; Lorenz and Domzalski, 2020; Serner et al., 2020). For example, Mendiguchia et al. proposed a multifactorial criteria-based progressive algorithm for hamstring injury treatment, which has been adapted in this case (Mendiguchia et al., 2017). Our outcomes demonstrate similar effectiveness, suggesting the potential advantages of a structured and criteria-based approach for rectus femoris strains.

At the first physical examination, the patient showed significant physical impairment due to important limitations to the AROM of the knee flexion and the hip flexor strength. Following a program based on specific training of the rectus femoris strength, lumbopelvic stabilization, hip flexors flexibility, and running technique exercises, the patient reported pain when performing his daily tasks only during the first 2 weeks after the injury. The patient was able to run at 10 km/h 25 days after the injury. The return to playing recreational soccer without discomfort during functional activities occurred 6 weeks after the injury due to the soccer team demands.

This design process takes into account the biological tissue repair principles (which include the stages of inflammation, proliferation, and remodeling) (Jarvinen et al., 2014), the injury mechanism (i.e., sprinting mechanics) (Garrett et al., 1987; Novacheck, 1998), and multiple risk factors related to muscle injury (Orchard, 2001). Initially, the focus was on reducing inflammation and pain, followed by progressively loading the muscle to stimulate collagen synthesis and muscle fiber regeneration, ultimately aiming to restore full function through graduated exercises. In addition, this clinical decision-making approach depends on the outcome of the previous step and is based on an individualized response to progress in difficulty according to the rectus femoris recovery (Schoenfeld et al., 2019). The clinical management was based on different risk factors and biological muscle repair. The proposed approach was sequenced with progress in exercise difficulty/intensity. For example, rectus femoris strength training was performed through its muscular actions (hip flexion and knee extension) (Hasselman et al., 1995).

Throughout the 6 weeks of treatment, the right knee flexion range of motion and the right hip flexor strength improved. In addition, the hip and knee extensor strength of both limbs improved. At clinical discharge, 6 weeks after the injury, the patient achieved his goal of returning to play recreational soccer. This case demonstrates that the return to sport timetable for recreational soccer players may be controlled by the criteria of progression according to the functional and clinical limitations throughout the entire treatment. Therefore, a specific prevention program focused on treating hip flexion strength deficits should be performed. This should serve as a warning in the management of muscle injuries, emphasizing the importance of maintaining specific exercises to prevent deficits and asymmetries in muscle strength (Piccoli et al., 2018).

To the authors’ knowledge, while several studies have proposed conservative treatments for rectus femoris muscle injuries, few have provided a detailed, criteria-based rehabilitation protocol specific to this injury. This report contributes by offering a structured approach that can be referenced in future research and clinical practice (Bogwasi et al., 2023). The results showed in this study should be interpreted with caution as this is a case report. This novel criteria-based algorithm rehabilitation program for a grade 1 rectus femoris strain could serve as a reference for future studies and should be tested in randomized controlled trials with soccer players.

### Patient perspective

The patient was grateful for being able to return to play within a reasonable period of time without having recurrences of his injury.

### Informed consent

The patient provided verbal consent to publish his data. As only one patient was described and standard-of-care clinical services were provided, the medical center required no formal Institution Review Board approval.

## Conclusion

The present case report study showed a 6-week-criteria-based progressive rehabilitation program implemented on a recreational soccer player diagnosed with grade 1 rectus femoris strain, where the right knee flexion range of motion and the right hip flexor strength improved throughout the therapeutic intervention. Six weeks after the injury, the patient achieved his goal of returning to play recreational soccer, demonstrating that the return to sport timetable for recreational soccer players may be controlled by the criteria of progression according to the functional and clinical limitations throughout the entire treatment.

## Data Availability

The raw data supporting the conclusions of this article will be made available by the authors, without undue reservation.
